# Gastrointestinal Neoplasia Associated with Bowel Parasitosis: Real or Imaginary?

**DOI:** 10.1155/2011/234254

**Published:** 2011-11-30

**Authors:** Michael R. Peterson, Noel Weidner

**Affiliations:** Department of Pathology, UCSD, 200 W Arbor Drive, MC8720, San Diego, CA 92126, USA

## Abstract

Several parasitic species are well known to have carcinogenic properties, namely; *Schistosoma hematobium* (squamous cell carcinoma of the bladder) and the liver flukes *Opisthorchis* and *Chlonorchis* (cholangiocarcinoma). A large number of parasites are known to colonize the gastrointestinal tract. We sought to review the evidence that implicates these parasites in gastrointestinal neoplasia. *Schistosoma japonicum*, which is endemic primarily in east Asia, has been shown in multiple studies to convey a mildly increased risk of colorectal adenocarcinoma. The data supporting a causative role for *Schistosoma mansoni* in colorectal or other neoplastic processes are less convincing, limited primarily to small case-control studies and case series. Reports of possible associations between other gastrointestinal parasites (e.g., *E. histolytica *and *A. lumbricoides*) and neoplasia may be found in the literature but are limited to individual cases. We conclude that, other than *S. japonicum* and to a lesser extent *S. mansoni*, there is little evidence of an association between gastrointestinal parasites and neoplasia.

## 1. Introduction

A wide variety of parasites are known to cause disease in the human gastrointestinal tract, including some species that are very prevalent over a large geographical area. Human parasites are traditionally divided into two broad groups, protozoa and helminths. The phylum protozoa includes a number of gastrointestinal parasites, with some notable members such as *Entamoeba histolytica, Giardia lamblia*, *Cryptosporidia*, and *Trypansoma cruzi*. The multi cellular helminths are further divided into three groups, cestodes/tapeworms (e.g., *Taenia solium* and *Diphyllobothrium latum*), nematodes/round worms (e.g., *Ascaris lumbricoides*, *Strongyloides stercoralis*, and *Enterobius vermicularis*), and trematodes/flukes (e.g., *Schistosoma japonicum* and *Schistosoma mansoni*).

There has long been scientific interest in exploring the possibility of infectious causes of cancer, including bacterial, viral, and parasitic causes. Mathematical modeling has estimated that approximately 16% of cancers throughout the world may be attributable to infection [1]. The fraction of this that is attributable to parasitic infection is currently unknown. Historically, one of the first proposed links between parasitosis and cancer garnered Dr. J. A. G. Fibiger the Nobel Prize in 1926 for his work demonstrating that mice infected with the nematode *Spiroptera* later developed stomach cancer. This work was later debunked on at least two levels. It was shown that the risk of “cancer” was only seen in a specific strain of mice, and later review actually challenged whether the lesions in question were truly carcinomatous in nature or just hyperplastic [2]. Despite this inauspicious beginning, several parasites have subsequently been definitively shown to have carcinogenic properties. Associations between *S. hematobium* and squamous cell carcinoma of the bladder and the liver flukes (*Opisthorchis* and *Clonorchis*) and cholangiocarcinoma have been well documented for decades. Thus, there is a body of evidence that links specific parasites to nongastrointestinal malignancies. Despite the fact that the gastrointestinal tract is the main site of infection for many parasites, there has, to our knowledge, been no review that explores the possible association between parasites of the gastrointestinal tract and subsequent neoplasia. 

## 2. Schistosomiasis

Schistosomiasis is a major worldwide health problem, with an estimated 600 million at-risk people across 74 countries and directly affecting approximately 200 million people [3]. Schistosomes are dioecious trematodes that live in the blood stream of humans and other animals and utilize fresh water snails as an intermediate host. The clinical syndrome of schistosomiasis results primarily from the host response to the deposited eggs rather than to the adult parasites, eliciting a vigorous eosinophilic and granulomatous response, and eventually fibrosis. The severity of symptoms is related to the number of eggs deposited in the tissue. 

*S. hematobium* is endemic primarily in the Middle East, particularly in Egypt. There is a wealth of epidemiologic, experimental, and histopathologic data that link infection by *S. hematobium* with squamous cell carcinoma of the urinary bladder (reviewed in [4]). Most tellingly, as public health measures have caused a decline in *S. hematobium* in Egypt in the past decades, there has been a matching decline in squamous cell carcinoma of the bladder [5, 6]. The pathogenesis of *S. hematobium* is thought to be related to the chronic inflammatory response, resulting in increased numbers of macrophages and neutrophils that release reactive oxygen and nitrogen species that have the potential to cause DNA damage [4, 7]. Two main schistosome species, *S. mansoni* and *S. japonicum*, affect the gastrointestinal tract and liver. *S. mansoni* is distributed in Africa, the Middle East, and South America, while *S. japonicum* is found in China, the Philippines, and Indonesia. *S. japonicum* was previously endemic to Japan, but integrated control measures in place since the 1920s eventually eradicated schistosomiasis in the late 1970s [8]. Intestinal schistosomiasis provokes mucosal granulomatous inflammation, pseudopolyposis, ulceration, and superficial bleeding, primarily in the colon and rectum. Clinically, this is manifested most often as abdominal pain and diarrhea, although quite frequently intestinal schistosomiasis is clinically occult [9].

## 3. *Schistosoma japonicum*

### 3.1. Association with Colorectal Carcinoma

A link between *S. japonicum* and colorectal neoplasia has been debated for decades. The first reports on this topic began to appear in the English literature in the late 1950's [10]. Since this time, there have been a large number of patients reported individually or in groups with both colorectal carcinoma and evidence of infection by *S. japonicum* (reviewed in [11]). Case reports and small case series continue to appear in the literature with some regularity, including the authors of this paper [12–15]. Many of these case reports and case series originated in areas of endemic *S. japonicum*, including Japan, China, and the Philippines, or from immigrants from these areas. Although these types of investigations certainly provide tantalizing evidence, case reports and series alone cannot provide definitive evidence of a link between *S. japonicum* and colorectal neoplasia. 

A series of studies from China and Japan, published between 1983 and 2005, examined this topic from an epidemiological and geographical standpoint, which were reviewed by the International Agency for the Research of Cancer (IARC) in 1994 [11, 16–20]. These studies analyzed the correlation between mortality due to colorectal carcinoma and that due to schistosomiasis in provinces of China with variable levels of *S. japonicum* prevalence. Most found a positive and significant correlation between cancers of the colon and rectum and mortality due to schistosomiasis, although all are to some extent confounded by difficulties in controlling for influences such as diet and other causes of colorectal cancer. In Xu and Su 1984 [18], colorectal cancer mortality was compared to mortality due to *S. japonicum* in two provinces in eastern China with variable levels of schistosomiasis prevalence, including data over a three-year period (1977–1979). The prevalence of schistosomiasis in the studied areas ranged from 10% to 79%, as determined by skin testing and rectal biopsy. The investigators found a statistically significant positive correlation (Spearman's rank correlation coefficient, *r* = 0.68, *P* < 0.01) between mortality due to colorectal cancer and that due to *S. japonicum*. In comparison, there was no statistical relationship between mortality due to *S. japonicum* and that due to lung cancer.

One of the first case-control studies to examine the association between *S. japonicum* and colorectal cancer [21] found that schistosomal eggs were more likely to be found in patients with stomach and colorectal carcinoma than patients with benign disease. 1,458 resections of the gastrointestinal tract in Japan were reviewed, including 993 specimens with benign processes and 465 with malignant tumors. 16.8% of the specimens with malignancy were found to also show evidence of infection with *S. japonicum*, as compared to 9.6% of those with nonmalignant conditions, a difference that was statistically significant. Interestingly, patients with malignant tumors in the 40–49-year-old age range were much more likely to show evidence of *S. japonicum* infection. In Xu and Su 1984 [18], the investigators performed a case-control series, comparing 252 patients with colorectal cancer to matched lung cancer patients and healthy controls. This study found a statistically significant association between rectal cancer and history of schistosomal infection (odds ratio between 4.5 and 8.3 depending on the controls), but no significant association was found between colon cancer and schistosomal infection. In another more recent case-control series, 142 colon cancer patients were compared to 285 matched control patients in Sichuan, China [22]. Out of 142 colon cancer patients, 42 (30%) gave a history of previous *S. japonicum* infection, as compared to 14% of the control patients. From this data, the authors reported a statistically significant association between *S. japonicum* infection and colon cancer (odds ratio 3.3, *P* < 0.01). Their results indicate a fraction of disease attributable to schistosomiasis of 24% for colon cancer. The investigators did not specifically compare colon and rectal cancers with regards to schistosomiasis.

Because squamous cell carcinoma of the bladder is rare in the absence of *S. hematobium* infection, this link was relatively simple to conclusively establish. In contrast, adenocarcinomas that arise in patients with *S. japonicum* infection have few overt histopathological differences from those not associated with schistosomiasis. However, a few studies have noted subtle features that distinguish *S. japonicum*-associated colorectal carcinomas from nonschistosomal-associated cancers. Cancers arising in the context of *S. japonicum* infection tend to occur in young patients, with 57% of *S. japonicum*-associated cancers being found in the 21–40-year-old range, as compared to 39% of nonschistosomal-associated colon cancers [23]. One study [24] compared a series of 289 *S. japonicum*-associated colorectal carcinomas with 165 nonschistosomal associated colorectal carcinomas, with the intent of identifying transitional mucosal changes that might distinguish schistosomal-associated colorectal cancers. Cancers associated with *S. japonicum* tended to be more often well differentiated than controls (91.6% of schistosomal-associated colorectal cancers were well differentiated, as compared to 69.1% of nonschistosomal-associated cancers), while control cases were more frequently associated with adenomatous polyps. It was also noted in this study that cancers associated with *S. japonicum* tended to occur in younger patients (mean 40.3 years) than in nonschistosomal-associated cancers (mean 46.3), although no statistical significance was given. This group published a follow-up study in 1981 that examined a subset of the previously published group of *S. japonicum*-associated colorectal cancers [25]. This study reported that flat epithelial dysplasia was found in the 36 of 60 examined cases of schistosomal-associated colorectal cancers. Also noted was a change in the distribution of Paneth cells, similar to that seen in inflammatory bowel disease. The authors concluded that *S. japonicum*-associated colorectal cancers tended to have features similar to cancers arising in the context of inflammatory bowel disease, although this was not formally compared to a series of matched controls.

The precise pathogenesis of *S. japonicum*-associated colorectal cancers remains enigmatic, although, as discussed above, it appears that the chronic inflammatory response due to deposited schistosome ova plays an etiologic role [26]. This topic is difficult to study in a controlled setting, due to the complex and lengthy life cycle of schistosome species and the fact that the fresh water snails that act as intermediate hosts are problematic to cultivate in aquaria, particularly the species associated with *S. japonicum *[27]. Two studies have reported an increased risk of hepatocellular carcinoma in mice infected with *S. japonicum* (reviewed in [27]), but no similar animal model of *S. japonicum* driven colorectal carcinoma has been reported. Extracts of *S. japonicum* ova were tested for direct mutagenicity using the Ames test [28]. No evidence was found to suggest that ova release a mutagenic factor. A series from Egypt attempted to identify immunohistochemical and molecular features that may distinguish colorectal cancers that are associated with *S. mansoni* and nonschistosomal-associated colorectal cancers [29]. Twenty-four specimens with colorectal carcinoma with evidence of *S. mansoni* were compared to 45 nonschistosomal-associated colorectal cancers. There was no significant relationship between schistosomal status and p53 and c-myc expression. BCL-2 expression was found to be more often positive in *S. mansoni*-associated colorectal cancers (58.3% versus 33.3% in nonschistosomal colorectal cancers, *P* = 0.046). Another study attempted to identify characteristic mutations in p53 in schistosomal and nonschistosomal-associated rectal cancers [30]. In this study, twenty-two cases of rectal cancer with evidence of *S. japonicum* and twenty-two nonschistosomal rectal cancers were subjected to single strand conformational polymorphism testing for p53 mutations and subsequent sequencing. In schistosomal rectal cancers, thirteen p53 mutations were identified in ten cases, most of which were found to be base-pair substitutions, while thirteen mutations were found in nine nonschistosomal rectal cancers. A higher proportion of base-pair substitutions were found at CpG dinucleotides in *S. japonicum-*associated rectal cancers as compared to nonschistosomal rectal cancers, although this difference did not reach statistical significance. These findings led the authors to suggest that *S. japonicum* may release a directly genotoxic agent. In summary, the pathogenesis of *S. japonicum*-associated colorectal cancers may have some similarities to those seen arising in the setting of inflammatory bowel disease, but much remains unknown.

A few studies have endeavored to explore potential host factors that could predispose patients to develop squamous cell carcinomas of the bladder in the context of *S. hematobium*. For instance, glutathione S-transferase gene deletions were shown to be significantly more common in patients with *S. hematobium*-associated squamous cell carcinomas of the bladder [31]. Similarly, a specific arylamine N-acetyl transferase genotype was found to be more prevalent in patients with *S. hematobium*-associated squamous cell carcinoma of the bladder [32]. To date, no similar studies have been performed in *S. japonicum*-associated colorectal cancer. In the approaching era of relatively inexpensive and widely available high throughput gene sequencing, it will be fascinating to ascertain whether there are host factors that predispose patients to the development of colorectal carcinoma due to schistosomiasis. 

The IARC formally reviewed the association between *S. japonicum* and colorectal cancer in 1994, as part of a monograph examining the carcinogenic risks to humans of schistosomes, liver flukes, and *Helicobacter pylor*i [11]. The IARC found sufficient evidence to declare that *S. hematobium* and species of *Opisthorchis* and *Clonorchis* were definitely carcinogenic to humans. In this monograph, the IARC concluded that *S. japonicum* is *possibly* carcinogenic to humans. Since this monograph was published, only a handful of additional studies have been published on this topic, although at least one additional case control study published in 2005 did find epidemiological evidence again implicating *S. japonicum *as a causative agent in colorectal carcinoma [22].

### 3.2. Association with Cancers of Other Sites

In addition to the very probable link between *S. japonicum* and colorectal malignancy, there is evidence that implicates this parasite in hepatocellular carcinogenesis. An epidemiological study of 907 patients with chronic liver disease from Japan found that 19% of patients with nonneoplastic chronic liver disease and 51% of patient with hepatocellular carcinoma showed evidence of infection by *S. japonicum*, a difference that was found to be statistically significant [33]. In the same study, a 10-year prospective case-control study found that chronic hepatic schistosomiasis conveyed roughly the same risk for development of hepatocellular carcinoma as other causes of chronic hepatitis such as HCV, although note was made in this study that there were a high number of cases that showed coinfection by HCV and *S. japonicum*, possibly biasing the data. In a mouse model, it was demonstrated that *S. japonicum* infected mice were at significantly increased risk of hepatocellular carcinoma. Out of 61 mice with hepatic infection by *S. japonicum*, 48 had single or multiple hepatocellular carcinomas [34]. Similar findings have been shown in experimental models examining mice infected by *S. japonicum* and then also given carcinogenic agents [27].

Besides colorectal and hepatic neoplasia, there is no significant or consistent evidence of an association between *S. japonicum* and cancers of other sites. A few studies have peripherally addressed a potential link between *S. japonicum* and cancers of the stomach and esophagus. Several case series of gastric cancer associated with *S. japonicum* infection have been published from both China and Japan (reviewed in [11]). In one of the early epidemiologic studies [17], significant positive correlation was found in a single province between mortality due to *S. japonicum* and cancers of the stomach and esophagus, but results from other provinces were inconsistent. In a similar study [16], no positive correlation was found between prevalence of *S. japonicum* infection and cancers of the stomach and esophagus. Overall, there appears little but limited, anecdotal evidence to suggest that there is a link between gastric and esophageal neoplasia and infection by *S. japonicum*.

A few individual cases have been reported of other neoplasms with evidence of infection by *S. japonicum*, including gastrointestinal tumors. A case of a rectal carcinoid was described with evidence of *S. japonicum *in a 44-year-old Philippines-born female patient [35]. In a series of 26 goblet cell carcinoids of the appendix, three were found to also show *S. japonicum *eggs in the appendiceal wall [36]. A few case reports exist in the literature that also describe *S. japonicum *ova associated with nongastrointestinal tumors, such as squamous cell carcinoma of the skin [37], malignant schwannoma [38], bronchogenic carcinoma [39], and breast carcinoma [40]. Given the paucity of these reports, it is most likely that these represent extraordinary coincidences rather than evidence of a causative role for the parasite in carcinogenesis.

## 4. *Schistosoma mansoni*

### 4.1. Association with Colorectal Carcinoma

Patients, both individually and in small series, with evidence of both colorectal adenocarcinoma and *S. mansoni* infection have been reported for decades, primarily in the Middle East [12, 41–43]. However, unlike *S. japonicum*, there is a distinct lack of epidemiological evidence that links the geographical distribution of *S. mansoni* to cancers of any type, gastrointestinal or otherwise. In one series, a large group of patients in an area of endemic *S. mansoni* (Egypt) underwent lower endoscopy and biopsy. Of 638 patients with biopsies, 219 had histologic evidence of *S. mansoni *infection, while 26 had colorectal adenocarcinoma. However, only two patients in this series had evidence of both *S. mansoni* infection and colorectal adenocarcinoma, leading the authors to conclude that there was no causative role for *S. mansoni* in colorectal neoplasia [44]. In another more recent study, histopathologic and genetic changes in 40 schistosomal-associated colorectal cancers were compared to 20 colorectal cancers without evidence of schistosomiasis, all from an area of endemic *S. mansoni* (Egypt). The authors found that patients with schistosomal-associated colorectal cancers were younger (average age 35 versus 51 years for nonschistosomal-associated cancers, *P* < 0.05) and more often had multiple synchronous tumors (20% of schistosomal-associated cancers were synchronous as compared to 5% of nonschistosomal cancers, *P* < 0.05). Pathologically, colorectal cancers associated with schistosomiasis were more often mucinous (35% of schistosomal-associated with mucinous histology versus 10%, *P* = 0.02) and tended to present at high stage. Immunohistochemical staining revealed that significantly more schistosomal-associated colorectal cancers overexpressed p53 (80% of schistosomal-associated cancers versus 40%, *P* = 0.006). The authors speculated that p53 alterations may act as an inciting event in schistosomal-associated neoplasia [45].

As discussed in the above section on *S. japonicum*, a series of *S. mansoni*-associated colorectal cancers and nonschistosomal colorectal cancers were found to show no significant differences in p53 and c-myc immunohistochemical expression, but Bcl-2 was found to be significantly more likely to be expressed in *S. mansoni*-associated cancers [29]. A case series comparing 59 colorectal cancers in Egypt to a variety of control cases originating from the USA found *S. mansoni* ova in 32% of resection specimens, similar to the reported prevalence in Egypt in the general population [46]. This study found that *S. mansoni* ova were more commonly found in MSI-H colorectal cancers (50% of MSI-H cancers as compared to 22% of microsatellite stable cancers, *P* = 0.002). *S. mansoni* ova were also more frequent among patients whose tumors showed KRAS mutations, although this was seen only in a small number of patients (80% [4 of 5] of patients with KRAS mutations had *S. mansoni *ova versus 31% of the 42 patients with no evidence of KRAS mutations, *P* = 0.05).

Similar to *S. japonicum*, in 1994 the IARC formally reviewed the evidence for a causative link between *S. mansoni* infection and colorectal adenocarcinoma [11]. The authors of this monograph concluded that there is inadequate evidence to classify *S. mansoni* as a carcinogenic agent in humans.

### 4.2. Association with Cancers of Other Sites

*S. mansoni* ova have been reported as individual cases in a variety of other nongastrointestinal tumors, including prostatic adenocarcinoma [47], ovarian teratoma [48], uterine leiomyoma [49], squamous cell carcinoma of the uterine cervix [50], and inflammatory myofibroblastic tumor of the liver [51]. Other than these few case reports, there is no evidence to suggest that *S. mansoni *plays a causative role in the carcinogenesis of malignancies of other sites.

## 5. Other Parasites

A number of reports in the literature discuss the wide variety of parasites that may opportunistically infect immunosuppressed patients (reviewed in [52]). Similarly, a number of reports may be found in the literature of parasitic infections that were found to simulate malignant gastrointestinal neoplasms (reviewed in [53]). Much more rare are reports of parasitic infections that present concomitantly with a first-time diagnosis of gastrointestinal malignancy, that could potentially speak to a carcinogenic role of the parasitic infection. These reports are limited to individual reports and very small case series. A case report from Zaire describes a 53-year-old male with a sigmoid colon carcinoma with liver metastases. The tumor surface was found to be covered in *E. histolytica* trophozoites [54]. There are at least three case reports linking biliary ascariasis with extrahepatic cholangiocarcinoma and periampullary carcinoma. The first report described a 65-year-old male with obstructive jaundice. Imaging revealed linear structures suggesting biliary ascariasis. The common bile duct and pancreatic region later were found to have a coexistant periampullary carcinoma [55]. Two women in rural Malaysia presenting with obstructive jaundice were both found to have imaging evidence of ascariasis in the common bile duct. Both women underwent surgery, revealing masses in the extrahepatic biliary tract that proved to be cholangiocarcinoma, as well as grossly identified worm parts in the biliary system [56]. The final reported case involved a 42-year-old male with obstructive jaundice and imaging evidence of biliary ascariasis. The worm was removed endoscopically, but jaundice persisted due to a periampullary mass that was found to represent a well-differentiated periampullary adenocarcinoma upon resection [57]. Colonic anisakiasis has been reported in conjunction with colorectal cancer twice, although one of these actually showed anisakiasis of the ascending colon in the context of a sigmoid colon cancer [58, 59]. Giardia infection has been documented in pancreatic adenocarcinomas twice. The first was reported in a 59-year-old Japanese woman with a hetergenous enhancing mass of the pancreas. ERCP was performed, with brush cytology that revealed *G. lamblia* trophozoites but no cancer. A follow-up EUS-FNA a month later did identify adenocarcinoma, and a subsequent pancreaticoduodenectomy revealed a ductal adenocarcinoma [60]. The second report was that of a 69-year-old Japanese male with a radiologically identified pancreatic tail mass. ERCP with aspiration of the pancreatic juice revealed numerous *G. lamblia* trophozoites. Subsequent distal pancreatectomy and omentectomy revealed a well to moderately differentiated ductal adenocarcoma with metastasis to the omentum [61]. Given the paucity of these reports, one must conclude that these instances likely represent true coincidence and suggest no role for these parasites in subsequent neoplastic transformation.

In addition, a few investigators have specifically addressed this potential link in larger studies and reviews for a few selected gastrointestinal anatomic sites and parasitic species. Infectious causes of esophageal neoplasia were reviewed by Eslick [62] who concluded that no parasites have been linked with esophageal cancers, although intriguingly noted that dogs are susceptible to a rare form of esophageal cancer following infection by the esophageal parasite *Spirocerca lupi*. A large group of patients with Chagas disease (*Trypansom cruzi*) in South America were examined, and no increased risk of gastrointestinal neoplasia was noted [63]. Although *Echinococcus* is not a gastrointestinal parasite, at least one published report found that Echinococcal hydatid cyst disease was significantly less prevalent than what would be expected given the prevalence in Turkey in a large group of cancer patients, leading the authors to conclude that this parasite may actually protect patients from neoplasia [64].

## 6. Conclusions

We have endeavored to review the literature that may demonstrate a causative role for parasites in gastrointestinal neoplasia, summarized in Table 1. The preponderance of evidence shows that *S. japonicum* infection of the colon and rectum conveys an increased risk for the development of adenocarcinoma. The molecular mechanisms that underlie this increased risk are not clear but are likely related to the long-term inflammatory response, similar to inflammatory bowel disease. The evidence linking *S. mansoni* infection and colorectal neoplasia is weaker, but at least one study does suggest that cancers arising in the setting of chronic *S. mansoni* infection have distinctive features, resembling cancers arising in the setting of inflammatory bowel disease [45].

Despite a scattering of case reports and small case series, there is no consistent or convincing evidence that links gastrointestinal neoplasia and any other parasite besides the schistosome species. Is there simply no significant relationship between most gastrointestinal parasites and neoplastic complications? Perhaps, but it is also possible that a weakly increased risk may be impossible to prove statistically in a parasite with low prevalence. Schistosomal infections are a very common parasitic infection in humans, resulting in a large infected population that facilitates epidemiological studies. Even in the well-studied carcinogenic parasites such as *S. hematobium* and the liver flukes, the increased risk of cancer is relatively low. It will require further very large epidemiologic studies to show a statistically increased risk of gastrointestinal neoplasia in parasites with low rates of prevalence, if one exists at all.

## Figures and Tables

**Table 1:** Summary of evidence linking gastrointestinal parasites and neoplasia.

Parasite	Cancer type	Site	Types of studies	Probability of causation of neoplasia
*S. japonicum*	Adenocarcinoma	Colon and rectum	Case-control studies, epidemiological studies, case series, and many case reports	Probable
*S. mansoni*	Adenocarcinoma	Colon and rectum	Case-control studies, case reports	Equivocal, weak association
*E. histolytica *	Adenocarcinoma	Colon	Rare case reports	Unlikely
*A. lumbricoides *	Adenocarcinoma	Biliary tract and ampulla	Rare case reports	Unlikely
*Anisakis*	Adenocarcinoma	Colon	Rare case reports	Unlikely
*G. lamblia*	Ductal adenocarcinoma	Pancreas	Rare case reports	Unlikely
